# Supplementary material for: Monitoring Behavior and Welfare of Cattle in Response to Summer Weather in an Arizona Rangeland Pasture Using a Commercial Rumen Bolus
Source: Animals (Basel). 2025 May 16;15(10):1448. doi: 10.3390/ani15101448 (PMC12108461; doi:10.3390/ani15101448)
Supplement: Supplementary file 1 [file animals-15-01448-s001.zip › animals-3562934-supplementary.pdf]

Supplementary Material

Table S1. Evaluation of SmaXtec and weather metrics (ambient temperature (AT), relative humidity (RH), wind speed, temperature–humidity index (THI), wet bulb globe temperature (WBGT) and solar load) using the 3 h data and the best models determined by the lowest Akaike Information Criterion (AIC) score. If none of the model coefficients were significant ($P > 0.05$), the best model is marked NS.

SmaXtec Metric	Weather Metric	Best Model	AIC
Activity Index	AT	Cubic	40,228
Activity Index	RH	Cubic	40,095.2
Activity Index	Wind	Linear	40,340.7
Activity Index	THI	Cubic	40,262.2
Activity Index	WBGT	Quadratic	40,334.2
Activity Index	Solar Load	Linear	40,110.3
Reticular Temperature	AT	NS	-
Reticular Temperature	RH	Linear	5981.9
Reticular Temperature	Wind	Linear	6041.5
Reticular Temperature	THI	Linear	6052.9
Reticular Temperature	WBGT	Quadratic	6017.7
Reticular Temperature	Solar Load	Linear	5941.8
Adjusted Reticular Temperature	AT	Cubic	−3828.6
Adjusted Reticular Temperature	RH	Cubic	−3851.2
Adjusted Reticular Temperature	Wind	Cubic	−3771.5
Adjusted Reticular Temperature	THI	Cubic	−3771.5
Adjusted Reticular Temperature	WBGT	Cubic	−3876.6
Adjusted Reticular Temperature	Solar Load	Linear	−3828.1
Rumination Index	AT	Quadratic	116,556.2
Rumination Index	RH	NS	-
Rumination Index	Wind	Cubic	116,545
Rumination Index	THI	Quadratic	116,556.3
Rumination Index	WBGT	Cubic	116,557.8
Rumination Index	Solar Load	NS	-

NS—not significant ($p > 0.05$).
